# Nonlinear Dynamics of Advancement Toward the ESI Top 1‰: Decomposition and Forecasting Evidence from an Emerging University

**DOI:** 10.3390/e28060652

**Published:** 2026-06-09

**Authors:** Fangqun Gao, Weiyan Hao, Yuntao Wu, Shubin Yang

**Affiliations:** 1 Library, Wuhan Institute of Technology, Wuhan 430205, China; 2 Hubei Key Laboratory of Intelligent Robot, Wuhan Institute of Technology, Wuhan 430073, China; 3 School of Electrical and Information Engineering, Wuhan Institute of Technology, Wuhan 430205, China

**Keywords:** Essential Science Indicators, ESI Top 1‰ scientometrics, threshold drift, nonlinear forecasting, bibliometric evaluation, competitive citation gap

## Abstract

Advancement toward the Essential Science Indicators (ESI) Top 1‰ is an important indicator of disciplinary competitiveness, but progress near this boundary is often nonlinear because the global threshold continues to move upward. This study develops a two-layer framework combining an Exponential Decay Model (EDM) for modeling rank trajectories and a Bivariate Logarithmic Difference Decomposition Model (BLDDM) for separating institutional citation growth from external threshold pressure. Using 13 bimonthly ESI update waves from March 2024 to March 2026, we analyze Chemistry, Engineering, and Materials Science at Wuhan Institute of Technology. The results show that the EDM outperforms a linear benchmark in all three disciplines, indicating an asymptotic pattern of advancement near the Top 1‰ boundary. The BLDDM further reveals substantial disciplinary heterogeneity: Engineering faces the strongest threshold pressure, whereas Chemistry is the most favorable near-term candidate for breakthrough. These findings suggest that ESI advancement should be understood as a moving-threshold process rather than a simple accumulation of citations.

## 1. Introduction

In the contemporary landscape of global research competition, the Essential Science Indicators (ESI) Top 1‰ benchmark has become a highly visible signal of disciplinary excellence and international research competitiveness [1,2]. For emerging universities, crossing this threshold is not merely symbolic; it is increasingly associated with research evaluation, resource allocation, institutional reputation, and strategic planning [1,3,4]. Yet a persistent paradox remains: many institutions continue to increase their citation counts while experiencing progressively slower gains in relative rank [5,6,7,8]. This mismatch suggests that advancement toward the ESI Top 1‰ is not a simple linear accumulation process, but rather a dynamic competitive process shaped by a moving external benchmark [9,10,11].

This issue is timely for two reasons. First, ESI-based indicators are increasingly used by universities, funding agencies, and research managers to monitor disciplinary competitiveness, allocate resources, and evaluate progress toward high-impact research performance. As a result, movement toward the ESI Top 1‰ has become not only a bibliometric outcome, but also a strategic management target for many emerging universities. Second, the rapid growth of global scientific output and citation accumulation makes elite bibliometric thresholds increasingly dynamic. A discipline may continue to increase its citation counts while making only limited progress in relative rank if the global benchmark rises at a similar or faster pace. This creates an urgent need for analytical tools that can distinguish genuine institutional progress from apparent progress that is partly offset by external threshold drift.

Existing bibliometric studies have provided important insights into university rankings, research competitiveness, highly cited publications, citation concentration, and research-front mapping [1,3,4,9,10,12]. Recent bibliometric applications have also demonstrated the value of bibliometric methods for identifying emerging themes and knowledge structures in fields such as artificial intelligence and digital business research [13,14]. However, much of this literature remains focused on static performance comparison, thematic mapping, or broad research-impact evaluation. Less attention has been paid to the dynamic process through which a near-threshold institution advances toward an elite ESI boundary when both institutional citations and the global cutoff are changing simultaneously.

To address this gap, this study develops a two-layer analytical framework. The first layer is an Exponential Decay Model (EDM), which is designed to capture the asymptotic slowdown of rank improvement near the elite threshold. The second layer is a Bivariate Logarithmic Difference Decomposition Model (BLDDM), which decomposes changes in the competitive citation gap into two interpretable components: institutional citation growth and external threshold pressure. Together, these models move the analysis beyond simple projection and toward a structural explanation of why some disciplines progress faster than others under different levels of competitive pressure.

Using 13 bimonthly ESI update waves from March 2024 to March 2026, this study examines three disciplines at Wuhan Institute of Technology: Chemistry, Engineering, and Materials Science. These three disciplines provide a useful comparative setting because all have entered the ESI Top 1% zone, yet all remain outside the Top 1‰ boundary and face different levels of external competitive pressure. The empirical analysis addresses three research questions:

1. Do ESI percentile rank trajectories near the Top 1‰ boundary follow a linear pattern or a nonlinear decelerating pattern?
2. To what extent is institutional citation growth offset by the upward movement of the global Top 1‰ threshold?
3. How sensitive are projected breakthrough windows to alternative assumptions about future threshold growth?

Accordingly, this paper makes three contributions. First, it introduces a nonlinear modeling approach for forecasting ESI rank trajectories under diminishing marginal returns. Second, it proposes a logarithmic decomposition framework that separates endogenous citation growth from exogenous competitive drift. Third, it links threshold volatility to projected breakthrough timing through scenario-based sensitivity analysis. Taken together, these contributions reframe ESI advancement not as passive ranking movement, but as a competitive-threshold process that can be measured, decomposed, and strategically managed.

## 2. Literature Review and Theoretical Framework

### 2.1. ESI-Based Evaluation and Bibliometric Research

Bibliometric indicators have become central tools for evaluating research performance, institutional competitiveness, and disciplinary development. Studies on university rankings and research competitiveness show that bibliometric indicators are closely associated with institutional visibility, global positioning, and research evaluation outcomes [1,3,4,15]. At the same time, research on highly cited publications and top-percentile outputs has emphasized that citation-based indicators are highly uneven and concentrated, especially near the upper tail of the citation distribution [9,10,12]. These findings suggest that movement toward elite bibliometric thresholds cannot be understood solely as an accumulation of publication volume or total citation counts.

The Essential Science Indicators (ESI) system is widely used to monitor disciplinary performance and identify highly cited research fields. In institutional research management, ESI indicators are often used to evaluate whether a discipline has entered the Top 1% or is approaching the more selective Top 1‰ boundary [16]. However, most institutional analyses of ESI advancement remain descriptive. They often report current rankings, citation counts, and distances from the threshold, but provide limited explanation of how the threshold itself changes over time. This limitation is important because the ESI Top 1‰ boundary is determined by the global distribution of citations and therefore changes as global scientific output and citation accumulation expand [2,6,17].

Recent bibliometric studies have also demonstrated the usefulness of bibliometric approaches for identifying research trends, thematic structures, and emerging knowledge domains. For example, bibliometric research on artificial intelligence has mapped major trends and emerging themes in leading scientific journals during the COVID-19 era [13]. Similarly, bibliometric review methods have been used to trace the evolution of customer engagement in digital business research and identify future research agendas [14]. These studies demonstrate the value of bibliometric analysis for understanding knowledge structures and research-front dynamics. Nevertheless, their primary focus is thematic mapping rather than modeling institutional advancement toward a moving elite threshold.

### 2.2. Limitations of Static and Linear Approaches

A key limitation in existing ESI-related assessment is the frequent reliance on static comparison or linear extrapolation [18]. Static comparison treats the target threshold as fixed and evaluates the institution by its current distance from that threshold. Linear extrapolation assumes that rank improvement proceeds at a constant marginal rate. Both approaches are convenient for institutional reporting, but they may be misleading when the global benchmark is changing and when marginal rank improvement slows near the elite boundary.

This limitation is especially relevant for near-threshold disciplines. A discipline may record steady citation growth while its relative rank improves only slowly if the global Top 1‰ threshold grows at a similar or faster pace. In such cases, institutional progress is partly absorbed by threshold drift. Moreover, as disciplines approach the elite zone, rank gains may become increasingly difficult because the leading part of the citation distribution is highly concentrated. Therefore, a more dynamic framework is needed to distinguish internal citation growth from external threshold pressure and to model nonlinear rank trajectories near the ESI Top 1‰ boundary.

### 2.3. Red Queen Effect and Matthew Effect as Theoretical Foundations

This study conceptualizes ESI advancement through the combined lens of the Red Queen Effect and the Matthew Effect. Originally developed in evolutionary theory, the Red Queen Effect describes a competitive environment in which agents must continuously improve merely to maintain their relative position [19,20,21]. In the context of ESI competition, the Red Queen Effect explains why institutional citation growth does not automatically translate into proportional rank improvement. The global Top 1‰ threshold is not fixed; it rises as global scientific output and citation accumulation continue to expand. Institutions near the elite boundary must therefore increase citation performance not only to advance, but also to prevent the moving benchmark from leaving them further behind.

The Matthew Effect provides a complementary explanation. In science, the Matthew Effect refers to cumulative advantage, whereby already visible and highly ranked actors tend to attract more attention, collaboration opportunities, and citations [22,23]. In the ESI context, this mechanism helps explain why the elite boundary may become increasingly steep. Citation advantages are concentrated among leading institutions, and their continuing accumulation raises the difficulty faced by emerging universities approaching the threshold. Thus, the Matthew Effect is not treated as a separate forecasting model in this study, but as a theoretical mechanism that informs the interpretation of nonlinear rank deceleration, threshold displacement, and cross-disciplinary differences in advancement difficulty.

Together, the Red Queen Effect and the Matthew Effect suggest that ESI advancement near the Top 1‰ boundary is a nonlinear competitive-threshold process. The Red Queen Effect explains the moving nature of the external benchmark, while the Matthew Effect explains the cumulative concentration of citation advantages near the top of the distribution. This combined perspective provides the theoretical foundation for the empirical framework developed in this study.

### 2.4. Research Gap and Analytical Framework

The literature reviewed above indicates three gaps. First, existing ESI-related assessments often focus on static rankings and descriptive indicators, while paying less attention to the movement of the global threshold. Second, many institutional forecasting practices rely on linear extrapolation, even though near-threshold rank trajectories may exhibit diminishing marginal returns. Third, although bibliometric studies have examined citation concentration and highly cited outputs, few have jointly modeled nonlinear rank trajectories and decomposed the citation gap into internal institutional growth and external threshold pressure.

To address these gaps, this study proposes a two-layer framework. The Exponential Decay Model (EDM) is used to model nonlinear rank trajectories and capture the asymptotic slowdown of advancement near the elite boundary. The Bivariate Logarithmic Difference Decomposition Model (BLDDM) is used to decompose changes in the competitive citation gap into institutional citation growth and external threshold pressure. This framework connects the theoretical logic of Red Queen threshold drift and Matthew-type cumulative advantage with measurable empirical indicators of ESI advancement.

## 3. Materials and Methods

### 3.1. Data and Research Design

All data utilized in this paper were derived from the historical database of Clarivate Essential Science Indicators (ESI), accessible at https://esi.clarivate.com/ (accessed on 15 March 2026). The official ESI platform updates its data every two months. The datasets adopted in this study are historical records archived by our research team at each official update wave. The observation window covers 13 bimonthly update waves from March 2024 to March 2026. Specifically, the update waves included March, May, July, September, and November 2024; January, March, May, July, September, and November 2025; and January and March 2026. Although the observation window is relatively short for long-term nonlinear time-series forecasting, it is appropriate for the specific purpose of this study for three reasons. First, the 13 bimonthly waves form a continuous and internally consistent sequence of ESI historical updates, allowing rank trajectories, institutional citation accumulation, and global threshold movement to be observed under the same database structure. Second, the selected period corresponds to a critical advancement stage in which the three disciplines had already entered the ESI Top 1% range but had not yet crossed the Top 1‰ boundary, making it suitable for examining short-term nonlinear movement near an elite threshold. Third, the objective of the analysis is not to establish a universal long-term forecasting law, but to identify whether observed advancement follows a decelerating pattern and how this pattern differs across disciplines under a moving-threshold environment. The analytical sample focuses on three disciplines at Wuhan Institute of Technology: Chemistry, Engineering, and Materials Science. These disciplines were selected because all three have already entered the ESI Top 1% range but have not yet crossed the Top 1‰ threshold. This setting provides a suitable empirical context for examining rank advancement near an elite disciplinary boundary. The core variables include disciplinary ESI percentile ranks, institutional citation counts, and the corresponding global Top 1‰ citation thresholds for each discipline. Together, these variables allow us to jointly model rank dynamics, internal citation accumulation, and external competitive pressure. The percentile rank reflects the relative position of each discipline within the global ESI distribution, while the institutional citation count captures endogenous citation growth. The Top 1‰ citation threshold represents the external benchmark that a discipline must exceed to enter the elite zone. For each update wave, the three variables were recorded consistently from the same ESI historical snapshot. The institutional citation count was taken directly from the discipline-level institutional record of Wuhan Institute of Technology, while the Top 1‰ threshold was operationalized as the global citation cutoff required for entry into the corresponding ESI Top 1‰ field at that update wave. The competitive citation gap was then constructed by comparing the institutional citation count with the corresponding global threshold. The observed rank series reveal a common empirical pattern. All three disciplines show sustained improvement during the observation period, but the pace of improvement gradually declines over time. From March 2024 to March 2026, Chemistry improved from 3.010‰ to 1.815‰, Engineering from 3.045‰ to 2.042‰, and Materials Science from 3.217‰ to 2.259‰. At the same time, first- and second-difference diagnostics indicate that the rate of improvement in percentile rank decelerates over time. This pattern suggests that a simple linear trend is unlikely to provide a credible extrapolation near the ESI Top 1‰ boundary.

### 3.2. Model Selection Strategy

Because the dataset contains only 13 observations for each discipline, the analysis was designed as a short-window, near-threshold modeling exercise rather than a long-term forecasting study. Model selection therefore prioritized parsimony, predictive stability, and extrapolation credibility. We deliberately avoided treating high in-sample fit as sufficient evidence of model validity, because flexible nonlinear models may overfit short time series and generate unstable long-range projections. Accordingly, model evaluation combined goodness-of-fit statistics with leave-one-out cross-validation, residual diagnostics, and substantive plausibility of the projected trajectories.

First, model fit was assessed using the Akaike Information Criterion (AIC) and the Bayesian Information Criterion (BIC). Both criteria balance goodness of fit against model complexity, with lower values indicating better model performance. Second, predictive stability was evaluated through leave-one-out cross-validation using the root mean squared error (LOOCV-RMSE). This procedure is appropriate for small-sample settings because it tests the model’s ability to predict each observation when that observation is excluded from estimation. Third, candidate models were screened for extrapolation plausibility. Models that produced unstable or substantively implausible out-of-sample trajectories were excluded, even if they showed strong in-sample fit.

The broader model comparison considered exponential decay, linear regression, logarithmic regression, power-function regression, and random-walk baselines. More flexible approaches, including Gaussian process regression, high-order polynomial regression, support vector regression, and Bayesian ridge regression, were also examined.

To provide empirical support for model selection, we report the performance of all candidate models in Supplementary Table S1. The comparison includes the exponential decay model, linear regression, logarithmic regression, power-function regression, random-walk baseline, Gaussian process regression, high-order polynomial regression, support vector regression, and Bayesian ridge regression. For each model, we report model-fit and predictive-performance indicators, including $R^{2}$, AIC, BIC, in-sample RMSE, and LOOCV-RMSE where applicable.

The results show that the Exponential Decay Model provides the best overall balance between goodness of fit, small-sample predictive stability, parsimony, and substantive interpretability. Although some flexible models achieve strong in-sample fit, their extrapolation behavior is less stable under the short 13-wave observation window. Overall, the comparison supports the use of the exponential decay specification as the preferred model for the main analysis.

### 3.3. Exponential Decay Model (EDM)

To capture the non-linear slowdown as subjects approach the elite zone, we employ:

(1) $$y\left(t\right)=a+b\cdot e^{-ct}+\varepsilon$$

where y(t) denotes the ESI percentile rank at time *t*, *a* is the asymptotic floor, *b* captures the initial distance from that floor, and *c* is the resistance coefficient governing the speed at which improvement decays over time. This specification is appropriate when early improvements are relatively rapid but subsequent gains become progressively smaller as the discipline approaches a competitive boundary. In substantive terms, the model interprets elite advancement as a process of diminishing marginal returns rather than constant-speed movement. Compared with a linear regression model, the EDM is better suited to capturing asymptotic rank trajectories in the high-ranking zone.

### 3.4. Bivariate Logarithmic Difference Decomposition Model (BLDDM)

Forecasting rank trajectories alone does not explain whether improvement is driven primarily by institutional effort or by changes in the external environment. To separate these mechanisms, this study introduces the Bivariate Logarithmic Difference Decomposition Model (BLDDM). Let *C* denote institutional citations and *T* denote the global Top 1‰ citation threshold. We define the logarithmic competitive gap as

ln(T/C)=ln(T)−ln(C)

A reduction in this gap indicates movement toward the Top 1‰ boundary. Taking first differences yields:

Δln(T/C)=Δln(T)−Δln(C)

which can be rearranged as

Netgapreduction=Δln(C)−Δln(T)

This identity decomposes changes in the competitive gap into two components. The first component, Δln(C), represents institutional citation growth. The second component, Δln(T), represents external threshold pressure. When institutional citation growth exceeds the threshold growth, the discipline moves closer to the Top 1‰ boundary. Conversely, when threshold growth absorbs a large share of institutional gains, progress toward the boundary slows despite continued citation accumulation. The BLDDM therefore provides an interpretable measure of Red Queen resistance by quantifying how much of a discipline’s internal citation growth is offset by the expansion of the global benchmark.

### 3.5. Scenario-Based Sensitivity Analysis

To evaluate the robustness of breakthrough projections, this study extends the EDM results through scenario-based sensitivity analysis. Three threshold-growth scenarios are considered: optimistic, baseline, and pessimistic. The baseline scenario assumes that the current growth rates of institutional citations and global thresholds remain unchanged. The optimistic scenario assumes a moderation in threshold growth, while the pessimistic scenario assumes an acceleration of external competition.

For each discipline, projected breakthrough timing is estimated under the three scenarios. Given the short observation window, these projections should be interpreted as conditional, scenario-based estimates rather than deterministic long-term forecasts. The purpose of this analysis is not to predict the exact date of future breakthrough, but to assess how sensitive the expected breakthrough window is to changes in the external threshold-growth rate. This is particularly important because the Top 1‰ boundary is not static; it changes as global citation accumulation continues. Scenario analysis therefore helps identify disciplines whose projected advancement is relatively stable and disciplines whose breakthrough timing is highly vulnerable to threshold volatility.

### 3.6. Validation Logic

The methodological framework is validated at two levels. First, the EDM is assessed through comparative fit statistics and residual diagnostics. The model is considered credible when it improves upon the linear benchmark in terms of AIC, BIC, and LOOCV-RMSE, and when its residuals do not show obvious systematic patterns. Second, the BLDDM is evaluated through interpretability and internal consistency. Because the decomposition is derived from a logarithmic identity, it provides a transparent accounting framework for separating institutional citation growth from external threshold pressure.

Together, these validation procedures ensure that the proposed framework is not only statistically appropriate for the observed data, but also substantively meaningful for understanding advancement toward the ESI Top 1‰.

Before turning to the model results, we first visualize the evolution of the global Top 1‰ citation thresholds during the observation period. This descriptive evidence provides the empirical basis for treating the Top 1‰ boundary as a moving benchmark rather than a fixed target.

**Figure 1:**
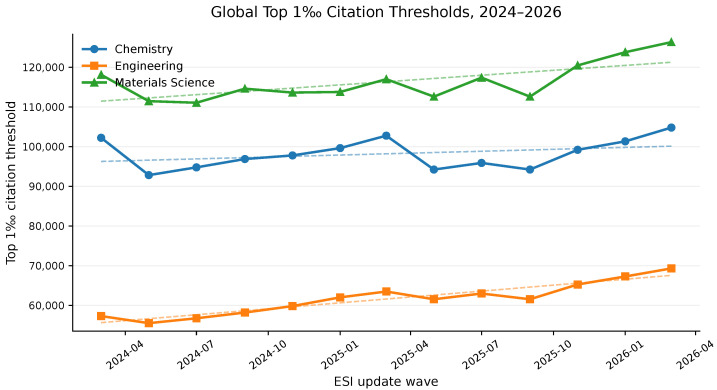
Dynamics of global Top 1‰ citation thresholds from March 2024 to March 2026. Solid lines show the observed thresholds, and dashed lines with the same colors represent the corresponding linear trend lines. The upward trajectories indicate that the benchmark for entry into the ESI Top 1‰ is continuously rising, thereby creating a moving competitive target for different disciplines.

As shown in Figure 1, the threshold trajectories exhibit a clear upward tendency across the three disciplines, supporting the need to account for external threshold drift in the subsequent forecasting and decomposition analyses.

## 4. Results

### 4.1. Model Fit and Validation

We first evaluated whether the Exponential Decay Model (EDM) provides a more credible representation of ESI rank improvement than the conventional Linear Regression Model (LRM). Across Chemistry, Engineering, and Materials Science, the observed trajectories show a common pattern: percentile ranks improve continuously over time, but the pace of improvement gradually slows as the disciplines approach the Top 1‰ boundary. This pattern is consistent with diminishing marginal returns in elite-threshold competition.

Model performance was first compared using $R^{2}$, AIC, BIC, and LOOCV-RMSE. The full comparison across all candidate models is reported in Supplementary Table S1. As a focused comparison between the preferred nonlinear specification and the conventional linear benchmark, Table 1 reports the EDM and LRM results for the three disciplines.

For Chemistry, the EDM increases $R^{2}$ from 0.975 to 0.991 and reduces LOOCV-RMSE from 0.0709 to 0.0503. For Engineering, the improvement is even more pronounced: $R^{2}$ increases from 0.983 to 0.998, while LOOCV-RMSE decreases from 0.0483 to 0.0246. For Materials Science, the EDM also achieves a higher $R^{2}$ and lower prediction error than the LRM. These results indicate that the EDM provides both better in-sample fit and stronger small-sample predictive stability.

To improve the transparency and reproducibility of the nonlinear specification, Table 2 reports the estimated EDM parameters for the three disciplines. The asymptotic floor parameter *a* indicates the fitted lower bound approached by the rank trajectory, *b* represents the initial distance from that floor, and *c* is the resistance coefficient governing the rate at which marginal rank improvement decelerates over time. The positive estimates of *c* across all three disciplines support the interpretation that ESI rank improvement follows a decelerating process near the elite boundary.

**Figure 2:**
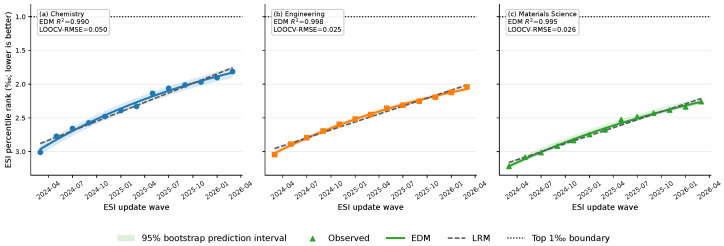
Observed and fitted ESI rank trajectories under the EDM and LRM. Panels (**a**–**c**) correspond to Chemistry, Engineering, and Materials Science, respectively. In panel (**a**), the blue circles and blue solid line represent the observed and EDM-fitted rank trajectory for Chemistry; in panel (**b**), the orange squares and orange solid line represent the observed and EDM-fitted rank trajectory for Engineering; and in panel (**c**), the green triangles and green solid line represent the observed and EDM-fitted rank trajectory for Materials Science. The grey dashed lines denote the corresponding LRM fits, the shaded bands indicate the 95% bootstrap prediction intervals, and the dotted horizontal line marks the ESI Top 1‰ boundary. The figure shows that the EDM better captures the decelerating pattern of rank improvement near the Top 1‰ boundary.

The fitted trajectories are presented in Figure 2. The LRM captures the general direction of rank improvement, but it imposes a constant rate of advancement. By contrast, the EDM better follows the observed deceleration in rank improvement, especially in the later update waves. This difference is substantively important because disciplines near the Top 1‰ boundary are unlikely to continue improving at a constant linear pace. Instead, the evidence suggests that advancement becomes increasingly resistant as the threshold is approached.

**Figure 3:**
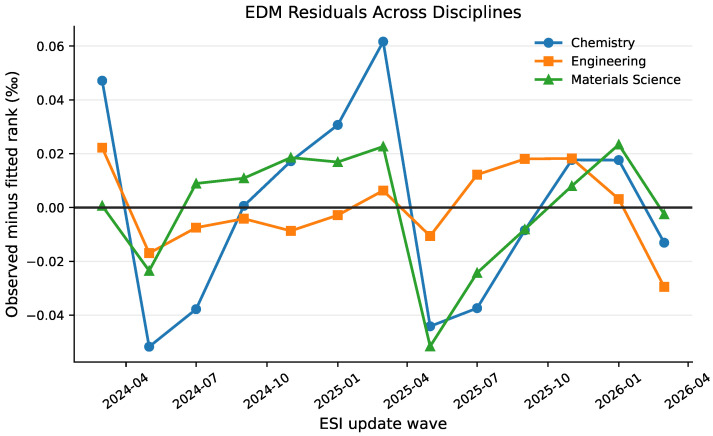
Residual analysis of the EDM across the three disciplines. The residuals are distributed around zero without obvious systematic structure, supporting the adequacy of the nonlinear specification.

Residual diagnostics further support the adequacy of the EDM. As shown in Figure 3, residuals are distributed around zero without obvious systematic structure. This suggests that the EDM captures the main nonlinear pattern in the observed rank trajectories and does not leave a clear directional bias in the fitted values.

### 4.2. Quantifying Red Queen Resistance

After establishing the superiority of the EDM for modeling rank trajectories, we used the BLDDM framework to quantify the extent to which institutional citation growth is offset by external threshold expansion. This analysis provides an empirical measure of Red Queen resistance: the stronger the threshold expansion relative to institutional citation growth, the more difficult it becomes for a discipline to convert citation gains into rank advancement.

As reported in Table 3, the three disciplines exhibit substantial heterogeneity in their competitive environments. Engineering records the highest annual institutional citation growth, at 30.2%, but it also faces the fastest annual global threshold growth, at 9.5%. As a result, its threshold displacement rate reaches 31.5%, indicating that nearly one-third of its internal citation-growth gains are offset by the expansion of the global Top 1‰ threshold. This makes Engineering the discipline with the strongest Red Queen resistance among the three cases.

Chemistry presents a more favorable competitive profile. Although its annual institutional citation growth is slightly lower than that of Engineering, at 23.9%, its annual global threshold growth is only 1.2%. Consequently, its threshold displacement rate is only 5.2%, meaning that most of its internal citation gains are retained as effective progress toward the Top 1‰ boundary. Materials Science occupies an intermediate position, with annual institutional citation growth of 22.5%, annual threshold growth of 3.4%, and a threshold displacement rate of 15.0%.

**Figure 4:**
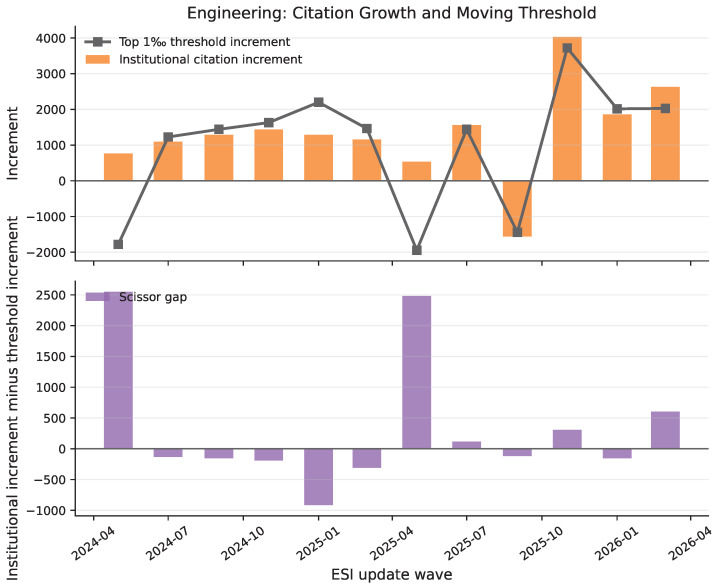
The “scissor gap” between institutional citation growth and global threshold expansion in Engineering. The figure illustrates how gains in institutional citations are partially offset by fluctuations in the external benchmark.

The “scissor gap” in Engineering is further illustrated in Figure 4. Although institutional citation increments remain positive, the global threshold also expands substantially during several update intervals. This pattern explains why strong citation growth does not necessarily produce proportional rank improvement. In other words, Engineering is advancing, but the external benchmark is also moving rapidly.

**Figure 5:**
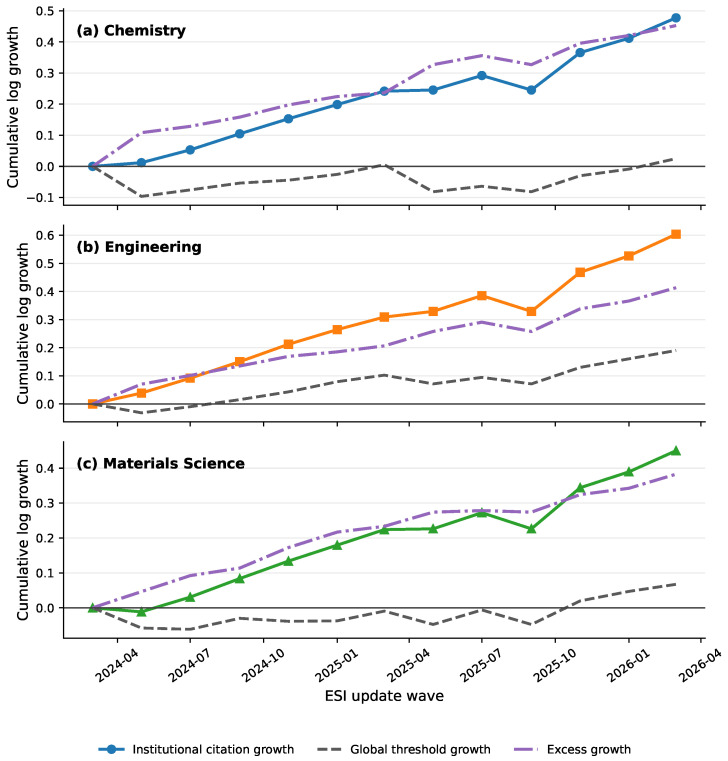
Institutional citation growth, global threshold growth, and excess growth across the three disciplines. Panels (**a**–**c**) correspond to Chemistry, Engineering, and Materials Science, respectively. Blue circles/line, orange squares/line, and green triangles/line represent the cumulative institutional citation growth for Chemistry, Engineering, and Materials Science, respectively. The grey dashed lines indicate cumulative global threshold growth, and the purple dash-dotted lines indicate cumulative excess growth, defined as institutional citation growth minus global threshold growth. The figure compares the balance between endogenous citation gains and exogenous threshold pressure over time.

Figure 5 compares institutional citation growth, global threshold growth, and excess growth across the three disciplines. The contrast between Chemistry and Engineering is especially clear. Chemistry benefits from a relatively slow-moving threshold, allowing institutional gains to translate more directly into competitive progress. Engineering, by contrast, faces a more volatile and rapidly rising threshold, which reduces the effective impact of its citation growth.

**Figure 6:**
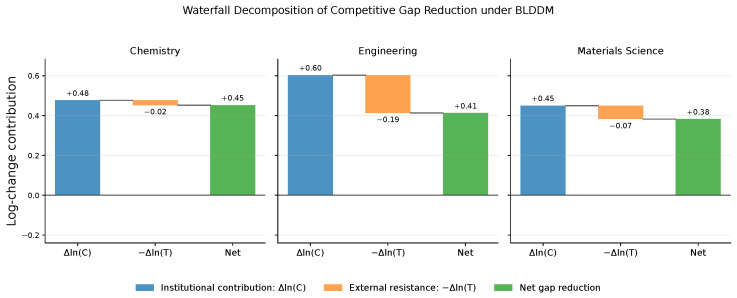
BLDDM decomposition of rank improvement into internal contribution, external resistance, and net improvement. The figure shows how much of the observed competitive progress is retained after accounting for threshold expansion.

The BLDDM decomposition in Figure 6 further clarifies this mechanism. Internal citation growth contributes positively to gap reduction in all three disciplines, but external threshold pressure offsets these gains to different degrees. Chemistry retains the largest share of its internal gains, Engineering loses the largest share to external threshold expansion, and Materials Science remains between the two. These findings confirm that the same level of institutional effort may produce very different ranking outcomes depending on the external competitive environment.

### 4.3. Projection of Breakthrough Windows

Finally, we estimated projected breakthrough timing under alternative threshold-growth scenarios. The purpose of this analysis is not to provide deterministic forecasts, but to evaluate how sensitive the expected entry into the ESI Top 1‰ is to changes in the growth rate of the global threshold.

As shown in Table 4, Chemistry is projected to achieve breakthrough earliest among the three disciplines. Under the optimistic, baseline, and pessimistic scenarios, its projected breakthrough timing remains March 2028. This stability reflects Chemistry’s relatively close current position and low threshold-growth pressure.

Engineering is projected to enter the Top 1‰ later and shows greater sensitivity to threshold conditions. Its projected breakthrough timing shifts from March 2029 under the optimistic scenario to July 2029 under the baseline scenario and November 2029 under the pessimistic scenario. This four- to eight-month delay under less favorable threshold conditions reflects the high volatility and fast growth of the Engineering threshold.

Materials Science shows the latest projected breakthrough window. Its estimated timing ranges from January 2030 under the optimistic scenario to March 2030 under the baseline scenario and May 2030 under the pessimistic scenario. Although its sensitivity to threshold variation is lower than that of Engineering, its larger structural gap delays its expected entry into the Top 1‰.

**Figure 7:**
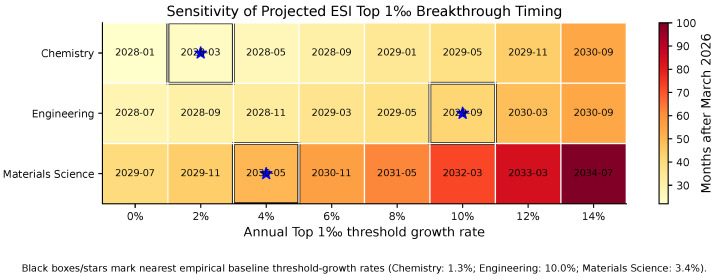
Sensitivity heatmap of projected breakthrough timing under alternative threshold-growth scenarios. The heatmap shows how the expected month of entry into the ESI Top 1‰ changes as the annual growth rate of the global threshold varies.

The sensitivity heatmap in Figure 7 provides a more detailed view of threshold-growth uncertainty. It shows that Chemistry remains relatively stable across a wide range of threshold-growth assumptions, whereas Engineering is highly sensitive to changes in threshold volatility. Materials Science is less volatile than Engineering but remains constrained by a longer distance from the Top 1‰ boundary. Overall, the projection results reinforce the central argument of this study: breakthrough timing is shaped not only by institutional citation accumulation, but also by the speed and volatility of the moving global benchmark.

After examining the sensitivity of projected breakthrough timing, we further summarize the cross-disciplinary strategic profiles using a normalized radar chart. This visualization provides a compact comparison of citation growth, threshold pressure, retained progress, and projected breakthrough proximity across the three disciplines.

**Figure 8:**
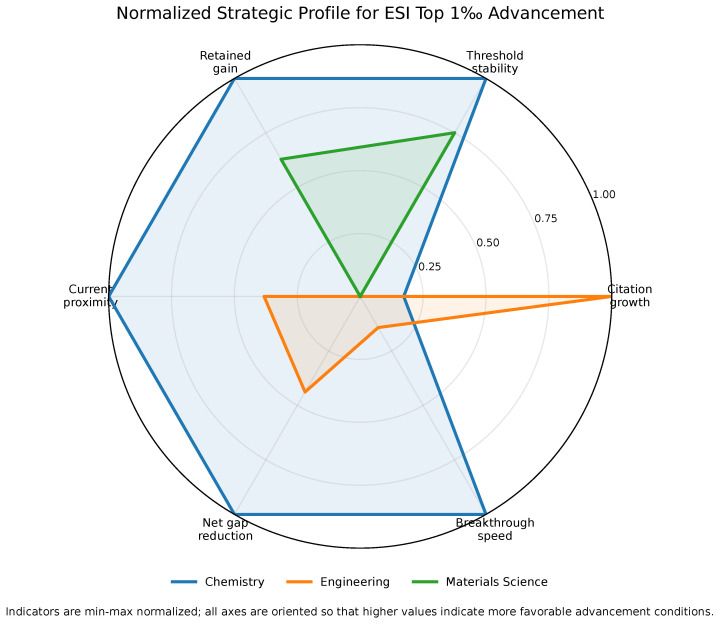
Normalized strategic profiles of the three disciplines. The radar chart compares institutional citation growth, inverse threshold pressure, retained progress, and breakthrough proximity after min–max normalization. Higher values indicate a more favorable strategic position.

As shown in Figure 8, Chemistry exhibits the most favorable near-term profile because of its low threshold pressure and strong retained progress. Engineering shows strong citation growth but is constrained by higher external threshold pressure, while Materials Science remains less favorable mainly because of its longer projected breakthrough window.

## 5. Discussion

The findings of this study indicate that advancement toward the ESI Top 1‰ boundary is not adequately explained by citation accumulation alone. Although all three disciplines examined in this study continued to increase their citation counts during the observation period, their movement toward the elite threshold differed substantially. This result supports recent bibliometric research showing that citation-based indicators are shaped by unequal citation distributions, ranking dynamics, and the concentration of highly cited outputs [9,10,12]. It also extends existing ESI-related and university-ranking studies by shifting attention from static performance comparison to the dynamic process of advancement under a moving global benchmark [1,3,4].

### 5.1. Theoretical Contributions: ESI Advancement as a Red Queen–Matthew Competitive Threshold Process

The first theoretical contribution of this study is to conceptualize ESI Top 1‰ advancement as a Red Queen–Matthew competitive threshold process. Existing bibliometric studies have shown that scientific impact is unevenly distributed and that top-percentile research outputs play a disproportionate role in research evaluation [9,10]. The present study adds that, for institutions approaching the ESI Top 1‰ boundary, this inequality has a dynamic implication: the threshold itself changes as leading institutions continue to accumulate citations.

The Red Queen mechanism explains why the Top 1‰ threshold is continuously moving upward as global citation accumulation expands. The Matthew Effect explains why this boundary may also become increasingly steep, because already visible and highly ranked institutions tend to accumulate additional citation advantages [22,23]. Together, these mechanisms imply that emerging universities must not only increase their own citation stock, but also overcome an elite boundary shaped by cumulative advantage at the upper end of the citation distribution.

This interpretation is consistent with the empirical evidence. The EDM results show that ESI rank improvement follows an asymptotic rather than linear trajectory, suggesting diminishing marginal returns near the elite boundary. The BLDDM results further show that part of institutional citation growth is offset by external threshold expansion. This offset can be interpreted not only as ordinary threshold drift, but also as a manifestation of Matthew-type cumulative advantage: as leading institutions continue to accumulate citations and visibility, the global benchmark becomes more difficult for emerging institutions to approach.

### 5.2. Methodological Contributions: From Static Comparison to Dynamic Decomposition

The second contribution is methodological. Much bibliometric research has used citation indicators to map knowledge domains, identify emerging themes, and evaluate research impact [13,14]. These approaches are valuable for understanding the structure and evolution of research fields. However, they are less suited to explaining how an institution advances toward a moving elite threshold over time. The present study addresses this issue by combining nonlinear trajectory modeling with logarithmic gap decomposition.

The comparison between the Exponential Decay Model and the Linear Regression Model shows that linear extrapolation may give a misleading impression of future progress when rank trajectories are slowing. The EDM provides a more appropriate representation of asymptotic advancement near the ESI Top 1‰ boundary. The BLDDM then adds an explanatory layer by separating institutional citation growth from external threshold pressure. Rank movement alone cannot reveal whether progress is driven by internal gains, favorable external conditions, or a combination of both. By decomposing the logarithmic citation gap, BLDDM provides a transparent way to evaluate how much institutional progress is retained after accounting for threshold drift.

This combined framework helps distinguish disciplines that may appear similar in rank trajectory but differ in competitive structure. For example, two disciplines may both improve over time, but one may do so under a relatively stable threshold while the other advances despite strong external pressure. Treating these cases as equivalent would obscure important differences in strategic difficulty. The proposed EDM–BLDDM framework therefore provides a more interpretable method for evaluating near-threshold ESI advancement.

### 5.3. Interpretation of Cross-Disciplinary Differences

The empirical results show substantial cross-disciplinary heterogeneity. Engineering has the highest institutional citation growth among the three disciplines, but it also faces the fastest growth in the global Top 1‰ threshold. As a result, a sizeable share of its internal citation gains is absorbed by external threshold expansion. From the perspective of the Matthew Effect, this suggests that the Engineering field may be characterized by stronger citation concentration and faster accumulation among leading institutions, which weakens the conversion of institutional citation growth into proportional rank advancement.

Chemistry presents a different pattern. Its threshold grows more slowly, allowing a larger proportion of institutional citation gains to translate into effective progress toward the Top 1‰ boundary. This explains why Chemistry is the most favorable near-term candidate for breakthrough despite not having the highest institutional citation growth rate. Materials Science lies between these two cases: its threshold pressure is lower than Engineering, but its remaining structural gap is larger than Chemistry. These differences confirm that ESI advancement depends not only on internal citation accumulation, but also on the external competitive environment of each discipline.

### 5.4. Practical Implications for Research Management

The practical implication of this study is that strategies for entering the ESI Top 1‰ should be discipline-specific rather than uniform. For disciplines already close to the elite boundary, further improvement depends less on publication volume alone and more on whether citation gains can exceed the rate of threshold expansion.

For Chemistry, the main advantage lies in its relatively favorable competitive environment. Its current position is closest to the Top 1‰ boundary, and its threshold growth is comparatively low. The strategic priority for Chemistry should therefore be to maintain citation momentum while concentrating resources on high-impact research areas, highly cited papers, and internationally visible collaborations. In this case, targeted improvement may be more effective than broad expansion.

Engineering presents a different challenge. Although its institutional citation growth is strong, the external benchmark is rising quickly. This means that ordinary citation growth may not be sufficient to close the gap. Engineering requires strategies that increase excess growth over the global threshold. Such strategies may include strengthening publication quality, improving the citation impact of existing outputs, promoting high-visibility research themes, and supporting collaborations that can raise the discipline’s citation premium.

Materials Science requires a longer-term strategy. Its threshold pressure is lower than that of Engineering, but its distance from the Top 1‰ boundary remains larger. For this discipline, both citation stock and citation quality matter. A feasible approach would combine continued growth in output with stronger attention to citation concentration, research direction selection, and the development of papers with sustained citation potential.

Overall, the results suggest that institutions near the ESI elite boundary should move beyond volume-oriented expansion. Increasing the number of publications may still contribute to citation growth, but it is unlikely to be sufficient when the external threshold is rising. A more effective strategy is to increase the citation premium of disciplinary output, namely the ability of publications to generate citation impact above the pace of global threshold growth.

## 6. Conclusions

This study examined the nonlinear dynamics of advancement toward the ESI Top 1‰ using 13 bimonthly ESI update waves from March 2024 to March 2026. Focusing on Chemistry, Engineering, and Materials Science at Wuhan Institute of Technology, the study proposed a two-layer analytical framework that combines the Exponential Decay Model with the Bivariate Logarithmic Difference Decomposition Model.

The results show that the Exponential Decay Model provides a better description of ESI rank trajectories than a linear benchmark across all three disciplines. This indicates that advancement near the Top 1‰ boundary follows an asymptotic pattern rather than a constant-rate linear trend. As the disciplines move closer to the elite zone, marginal improvements become more difficult to achieve.

The decomposition results further show that institutional citation growth and external threshold drift play different roles across disciplines. Chemistry benefits from a relatively slow-growing threshold and is therefore the most favorable near-term candidate for breakthrough. Engineering records strong citation growth but faces the strongest external resistance because of rapid threshold expansion. Materials Science shows a more gradual trajectory and is likely to require a longer development period before reaching the Top 1‰.

Theoretically, these findings suggest that advancement toward the ESI Top 1‰ boundary is shaped by the joint operation of Red Queen threshold drift and Matthew-type cumulative advantage, rather than by citation accumulation alone. The scenario analysis further confirms that breakthrough timing is sensitive to threshold conditions, particularly in Engineering. This finding highlights the importance of treating the ESI Top 1‰ boundary as a moving benchmark rather than a fixed target. For research management, the implication is clear: institutions approaching the elite threshold should focus not only on increasing citation counts, but also on improving the citation premium and competitive resilience of disciplinary output.

## 7. Limitations

This study has several limitations. First, the empirical analysis is based on 13 bimonthly ESI update waves, which is a short time series for nonlinear forecasting. Although this window is useful for identifying short-term nonlinear advancement patterns near the ESI Top 1‰ boundary, it is not sufficient to support strong claims about long-term forecasting stability. The projected breakthrough windows should therefore be interpreted as conditional scenario-based estimates rather than deterministic predictions. A longer observation window would allow more robust testing of parameter stability, structural change, and the persistence of nonlinear rank deceleration.

Second, the study focuses on a single institution. Wuhan Institute of Technology provides a useful case because several of its disciplines are close to the ESI Top 1‰ boundary, but the findings should be interpreted with caution when applied to institutions with different disciplinary portfolios, publication histories, or research management systems.

Third, the scenario analysis depends on assumptions about future institutional citation growth and global threshold growth. These assumptions are necessary for projection, but actual future trajectories may be affected by changes in publication volume, highly cited papers, disciplinary shocks, database updates, or shifts in global research activity.

Fourth, the current framework focuses on citation counts, percentile ranks, and threshold dynamics. It does not explicitly model article-level quality, collaboration networks, funding inputs, research team structure, or topic-level citation potential. These factors may influence the ability of a discipline to generate sustained citation impact.

Finally, highly cited outlier papers may affect short-term citation growth and projected breakthrough timing. Future research could extend the present framework by incorporating article-level citation distributions, collaboration indicators, and multi-institutional comparisons. Such extensions would provide a more detailed understanding of how disciplinary competitiveness evolves near elite bibliometric thresholds.

## Figures and Tables

**Table 1:** Comparison of model fit and predictive performance across the three disciplines.

Discipline	Model	$R^{2}$	AIC	BIC	LOOCV-RMSE
Chemistry	LRM	0.975	−71.03	−69.90	0.0709
Chemistry	EDM	0.991	−81.65	−79.95	0.0503
Engineering	LRM	0.983	−80.78	−79.65	0.0483
Engineering	EDM	0.998	−103.97	−102.28	0.0246
Materials Science	LRM	0.985	−81.85	−80.72	0.0448
Materials Science	EDM	0.995	−94.80	−93.10	0.0253

*Note:* EDM = Exponential Decay Model; LRM = Linear Regression Model; LOOCV-RMSE = Leave-One-Out Cross-Validation Root Mean Squared Error. Lower AIC, BIC, and LOOCV-RMSE values indicate better model performance.

**Table 2:** Estimated EDM parameters with standard errors and 95% confidence intervals.

Discipline	Parameter	Estimate	SE	95% CI	Interpretation
Chemistry	*a*	1.0763	0.3046	[0.4792, 1.6733]	Asymptotic floor of the rank trajectory
Chemistry	*b*	1.8864	0.2881	[1.3217, 2.4511]	Initial distance from the asymptotic floor
Chemistry	*c*	0.0767	0.0190	[0.0395, 0.1140]	Resistance coefficient governing deceleration
Engineering	*a*	1.4205	0.1346	[1.1567, 1.6842]	Asymptotic floor of the rank trajectory
Engineering	*b*	1.6020	0.1275	[1.3521, 1.8520]	Initial distance from the asymptotic floor
Engineering	*c*	0.0751	0.0096	[0.0563, 0.0939]	Resistance coefficient governing deceleration
Materials Science	*a*	1.3779	0.2870	[0.8153, 1.9404]	Asymptotic floor of the rank trajectory
Materials Science	*b*	1.8383	0.2766	[1.2962, 2.3804]	Initial distance from the asymptotic floor
Materials Science	*c*	0.0611	0.0135	[0.0347, 0.0875]	Resistance coefficient governing deceleration

*Note*: EDM = Exponential Decay Model; SE = standard error. The model is specified as $y(t)=a+be^{-ct}+\varepsilon$, where y(t) denotes the ESI percentile rank at time *t*. Confidence intervals are calculated from the parameter covariance matrix of the nonlinear least-squares estimation.

**Table 3:** Indicators of Red Queen resistance across Chemistry, Engineering, and Materials Science.

Metric	Chemistry	Engineering	Materials Science
Annual institutional citation growth	23.9%	30.2%	22.5%
Annual global threshold growth	1.2%	9.5%	3.4%
Threshold displacement rate	5.2%	31.5%	15.0%

*Note:* The threshold displacement rate refers to the proportion of internal citation-growth gains offset by the expansion of the global Top 1‰ threshold.

**Table 4:** Projected breakthrough timing under alternative threshold-growth scenarios.

Discipline	Optimistic	Baseline	Pessimistic
Chemistry	March 2028	March 2028	March 2028
Engineering	March 2029	July 2029	November 2029
Materials Science	January 2030	March 2030	May 2030

*Note:* The projected dates represent the expected bimonthly ESI update at which each discipline is forecast to enter the Top 1‰ under the specified scenario.

## Data Availability

The data used in this study were compiled from rolling Essential Science Indicators updates covering the period from March 2024 to March 2026. Reasonable requests for the processed dataset may be considered by the corresponding author.

## Supplementary Materials

The following supporting information can be downloaded at: https://www.mdpi.com/article/10.3390/e28060652/s1, Table S1: Performance comparison of all candidate models by discipline. Table S2: Average performance ranking of candidate models across the three disciplines.
