# Supplementary material for: Nonlinear Dynamics of Advancement Toward the ESI Top 1‰: Decomposition and Forecasting Evidence from an Emerging University
Source: Entropy (Basel). 2026 Jun 9;28(6):652. doi: 10.3390/e28060652 (PMC13298501; doi:10.3390/e28060652)
Supplement: Supplementary file 1 [file entropy-28-00652-s001.zip › entropy-4279835-supplementary.pdf]

Table S1. Performance comparison of all candidate models by discipline.

Discipline	Model	R^2	AIC	BIC	In-sample RMSE	LOOCV-RMSE	Extrapolation Behavior
Chemistry	Exponential Decay Model (EDM)	0.9906	–81.65	–79.95	0.0344	0.0502	Stable asymptotic trajectory; theoretically aligned with diminishing marginal rank improvement
	Linear Regression Model (LRM)	0.9752	–71.03	–69.90	0.0558	0.0709	Constant-rate extrapolation; simple but ignores deceleration near the elite boundary
	Logarithmic Regression	0.9506	–62.05	–60.92	0.0788	0.1055	Slowing trend; no explicit asymptotic floor for the ESI boundary
	Power-Function Regression	0.9186	–53.56	–51.87	0.1012	0.1465	Slowing nonlinear trend; less directly interpretable than EDM for threshold approach
	Random-Walk Baseline	0.8592	—	—	0.1143	0.1143	Naive no-structure baseline; uses previous observation as next prediction
	Polynomial Regression (degree 2)	0.9903	–81.21	–79.51	0.0349	0.0508	Flexible curvature; may overfit short windows and lacks a threshold mechanism
	Polynomial Regression (degree 3)	0.9907	–79.69	–77.43	0.0343	0.0609	High flexibility; higher risk of boundary oscillation in short samples
	Gaussian Process Regression (RBF)	0.9893	—	—	0.0367	0.0624	Flexible nonparametric fit; extrapolation is sensitive under short time series
	Support Vector Regression (RBF)	0.9971	—	—	0.0190	0.0707	Flexible nonlinear benchmark; weak parameter interpretability for bibliometric mechanisms
	Bayesian Ridge Regression (poly-2)	0.9903	—	—	0.0350	0.0514	Regularized polynomial benchmark; stable but less substantively interpretable than EDM
Engineering	Exponential Decay Model (EDM)	0.9976	–103.97	–102.28	0.0146	0.0246	Stable asymptotic trajectory; theoretically aligned with diminishing marginal rank improvement
	Linear Regression Model (LRM)	0.9832	–80.78	–79.65	0.0384	0.0483	Constant-rate extrapolation; simple but ignores deceleration near the elite boundary
	Logarithmic Regression	0.9549	–67.92	–66.79	0.0629	0.0912	Slowing trend; no explicit asymptotic floor for the ESI boundary
	Power-Function Regression	0.9291	–60.03	–58.34	0.0789	0.1211	Slowing nonlinear trend; less directly interpretable than EDM for threshold approach
	Random-Walk Baseline	0.8839	—	—	0.0879	0.0879	Naive no-structure baseline; uses previous observation as next prediction
	Polynomial Regression (degree 2)	0.9967	–100.07	–98.38	0.0169	0.0286	Flexible curvature; may overfit short windows and lacks a threshold mechanism
	Polynomial Regression (degree 3)	0.9993	–117.61	–115.35	0.0080	0.0128	High flexibility; higher risk of boundary oscillation in short samples
	Gaussian Process Regression (RBF)	0.9992	—	—	0.0084	0.0217	Flexible nonparametric fit; extrapolation is sensitive under short time series
	Support Vector Regression (RBF)	0.9992	—	—	0.0085	0.0523	Flexible nonlinear benchmark; weak parameter interpretability for bibliometric mechanisms
	Bayesian Ridge Regression (poly-2)	0.9967	—	—	0.0169	0.0286	Regularized polynomial benchmark; stable but less substantively interpretable than EDM
Materials Science	Exponential Decay Model (EDM)	0.9952	–94.80	–93.11	0.0207	0.0253	Stable asymptotic trajectory; theoretically aligned with diminishing marginal rank improvement
	Linear Regression Model (LRM)	0.9848	–81.85	–80.72	0.0368	0.0448	Constant-rate extrapolation; simple but ignores deceleration near the elite boundary
	Logarithmic Regression	0.9332	–62.64	–61.51	0.0771	0.1109	Slowing trend; no explicit asymptotic floor for the ESI boundary
	Power-Function Regression	0.9058	–56.17	–54.48	0.0915	0.1392	Slowing nonlinear trend; less directly interpretable than EDM for threshold approach
	Random-Walk Baseline	0.8950	—	—	0.0862	0.0862	Naive no-structure baseline; uses previous observation as next prediction
	Polynomial Regression (degree 2)	0.9955	–95.69	–93.99	0.0200	0.0250	Flexible curvature; may overfit short windows and lacks a threshold mechanism
	Polynomial Regression (degree 3)	0.9959	–94.88	–92.62	0.0191	0.0315	High flexibility; higher risk of boundary oscillation in short samples
	Gaussian Process Regression (RBF)	0.9959	—	—	0.0191	0.0306	Flexible nonparametric fit; extrapolation is sensitive under short time series
	Support Vector Regression (RBF)	0.9982	—	—	0.0126	0.0559	Flexible nonlinear benchmark; weak parameter interpretability for bibliometric mechanisms
	Bayesian Ridge Regression (poly-2)	0.9955	—	—	0.0200	0.0249	Regularized polynomial benchmark; stable but less substantively interpretable than EDM

Note: EDM = Exponential Decay Model; LRM = Linear Regression Model; LOOCV-RMSE = Leave-One-Out Cross-Validation Root Mean Squared Error; RBF = radial basis function. AIC and BIC are reported for parametric regression models. They are not reported for the random-walk baseline or for nonparametric/machine-learning models where the likelihood-based parameter count is not directly comparable. Lower AIC, BIC, and LOOCV-RMSE values indicate better model performance.

Table S2. Average performance ranking of candidate models across the three disciplines.

Rank	Model	Average R^2	Average LOOCV-RMSE	Average In-sample RMSE	Interpretation
1	Exponential Decay Model (EDM)	0.9945	0.0334	0.0232	Stable asymptotic trajectory; theoretically aligned with diminishing marginal rank improvement
2	Polynomial Regression (degree 2)	0.9942	0.0348	0.0240	Flexible curvature; may overfit short windows and lacks a threshold mechanism
3	Bayesian Ridge Regression (poly-2)	0.9942	0.0350	0.0240	Regularized polynomial benchmark; stable but less substantively interpretable than EDM
4	Polynomial Regression (degree 3)	0.9953	0.0351	0.0205	High flexibility; higher risk of boundary oscillation in short samples
5	Gaussian Process Regression (RBF)	0.9948	0.0382	0.0214	Flexible nonparametric fit; extrapolation is sensitive under short time series
6	Linear Regression Model (LRM)	0.9811	0.0547	0.0437	Constant-rate extrapolation; simple but ignores deceleration near the elite boundary
7	Support Vector Regression (RBF)	0.9982	0.0596	0.0134	Flexible nonlinear benchmark; weak parameter interpretability for bibliometric mechanisms
8	Random-Walk Baseline	0.8794	0.0961	0.0961	Naive no-structure baseline; uses previous observation as next prediction
9	Logarithmic Regression	0.9462	0.1025	0.0729	Slowing trend; no explicit asymptotic floor for the ESI boundary
10	Power-Function Regression	0.9178	0.1356	0.0905	Slowing nonlinear trend; less directly interpretable than EDM for threshold approach

Note: Models are ranked by average LOOCV-RMSE across Chemistry, Engineering, and Materials Science. Lower LOOCV-RMSE indicates better small-sample predictive performance.
